# Anthroposophic therapy for children with chronic disease: a two-year prospective cohort study in routine outpatient settings

**DOI:** 10.1186/1471-2431-9-39

**Published:** 2009-06-19

**Authors:** Harald J Hamre, Claudia M Witt, Gunver S Kienle, Christoph Meinecke, Anja Glockmann, Stefan N Willich, Helmut Kiene

**Affiliations:** 1 Institute for Applied Epistemology and Medical Methodology, Zechenweg 6, Freiburg, Germany; 2 Institute of Social Medicine, Epidemiology, and Health Economics, Charité University Medical Center, Berlin, Germany; 3 Paediatric Consultant, Community Hospital Havelhöhe, Berlin, Germany

## Abstract

**Background:**

Many children with chronic disease use complementary therapies. Anthroposophic treatment for paediatric chronic disease is provided by physicians and differs from conventional treatment in the use of special therapies (art therapy, eurythmy movement exercises, rhythmical massage therapy) and special medications. We studied clinical outcomes in children with chronic diseases under anthroposophic treatment in routine outpatient settings.

**Methods:**

In conjunction with a health benefit program, consecutive outpatients starting anthroposophic treatment for any chronic disease participated in a prospective cohort study. Main outcome was disease severity (Disease and Symptom Scores, physicians' and caregivers' assessment on numerical rating scales 0–10). Disease Score was documented after 0, 6, and 12 months, Symptom Score after 0, 3, 6, 12, 18, and 24 months.

**Results:**

A total of 435 patients were included. Mean age was 8.2 years (standard deviation 3.3, range 1.0–16.9 years). Most common indications were mental disorders (46.2% of patients; primarily hyperkinetic, emotional, and developmental disorders), respiratory disorders (14.0%), and neurological disorders (5.7%). Median disease duration at baseline was 3.0 years (interquartile range 1.0–5.0 years). The anthroposophic treatment modalities used were medications (69.2% of patients), eurythmy therapy (54.7%), art therapy (11.3%), and rhythmical massage therapy (6.7%). Median number of eurythmy/art/massage therapy sessions was 12 (interquartile range 10–20), median therapy duration was 118 days (interquartile range 78–189 days).

From baseline to six-month follow-up, Disease Score improved by average 3.00 points (95% confidence interval 2.76–3.24 points, p < 0.001) and Symptom Score improved by 2.41 points (95% confidence interval 2.16–2.66 points, p < 0.001). These improvements were maintained until the last follow-up. Symptom Score improved similarly in patients not using adjunctive non-anthroposophic therapies within the first six study months.

**Conclusion:**

Children under anthroposophic treatment had long-term improvement of chronic disease symptoms. Although the pre-post design of the present study does not allow for conclusions about comparative effectiveness, study findings suggest that anthroposophic therapies may play a beneficial role in the long-term care of children with chronic illness.

## Background

Chronic illness affects 15%–18% of children [1] and can lead to functional limitation, dependency on therapies and medication, poor school performance, and impaired quality of life [1-4]. Strategies to improve the outcome of chronic paediatric illness include enhanced healthcare provision [5] and special educational and behavioural interventions [6-8]. Many children with chronic disease also receive complementary therapies [9-13], sometimes provided by their physicians. In Germany, several physician-provided complementary therapies have been reimbursed by health insurance companies as part of special health benefit programs [14-17]. In most of these complementary therapies the physician is the active person, directly treating (e. g. giving acupuncture) or prescribing therapy (e. g. homoeopathic medications), while the patient has a predominantly passive role. Anthroposophic medicine (AM), a complementary system of medicine founded by Rudolf Steiner and Ita Wegman [18], includes active (AM art and eurythmy therapy) as well as passive therapy modalities (massage, medications).

In AM art therapy the patients engage in painting, drawing, clay modelling, music or speech exercises [19]. In addition to psychological effects (e. g. activation, emotive expression, dialogical communication with the therapist and with the artistic medium) [20,21], AM art therapy can induce physiological effects: e. g. AM speech exercises have effects on heart rate rhythmicity and cardiorespiratory synchronization which are not induced by spontaneous or controlled breathing alone [22,23].

Eurythmy therapy (Greek: eurythmy = "harmonious rhythm") is an active exercise therapy, involving cognitive, emotional, and volitional elements [24]. During eurythmy therapy sessions the patients are instructed to perform specific movements with the hands, the feet or the whole body. Eurythmy movements are related to the sounds of vowels and consonants, to music intervals or to soul gestures, e. g. sympathy-antipathy [25]. Eurythmy exercises have specific effects on heart rate variability [26]. Qualification as an AM art or eurythmy therapist requires six years' training according to an international, standardised curriculum.

Rhythmical massage therapy was developed from Swedish massage by Ita Wegman, physician and physiotherapist [27], and is practiced by physiotherapists with 1 1/2–3 years specialised training. In rhythmical massage therapy, traditional massage techniques (effleurage, petrissage, friction, tapotement, vibration) are supplemented by gentle lifting and rhythmically undulating, stroking movements, where the quality of grip and emphasis of movement are altered to promote specific effects [28].

AM medications are of mineral, botanical or zoological origin or are chemically defined substances [29].

Prior to prescription of AM medications or referral to AM therapies, AM physicians have prolonged consultations with the patients and their caregivers. These consultations are used to take an extended history, to address constitutional and psychosocial aspect of the patients' illness, to explore the patients' and caregivers' preparedness to engage in treatment, and to select optimal therapy for each patient [19,28].

AM is practiced in 67 countries [30]. Related to the AM approach is an educational philosophy implemented in more than 3,000 Waldorf Schools, kindergartens, and curative education centres worldwide [31,32]. Waldorf school attendance has been associated with a reduced risk for atopic disease [33,34], possibly mediated by effects on the intestinal microflora from restrictive use of antibiotics and antipyretics in childhood infectious disease [34] or from a vegetarian diet [35].

Observational studies suggest that AM therapies can have clinically relevant effects in children with chronic diseases [24,36-45]. All these studies were monocentric, and all but four studies [36,37,42,44] had a sample size of 30 or less AM patients. Here we present a pre-planned subgroup analysis of 435 children from a multi-centre long-term study of AM therapy [15].

## Methods

### Study design and objective

This is a prospective cohort study in a real-world medical setting. The study was part of a research project on the effectiveness, costs, and safety of AM therapies in outpatients with chronic disease (Anthroposophic Medicine Outcomes Study, AMOS) [15,46,47]. The AMOS project was initiated by a health insurance company in conjunction with a health benefit program. The present pre-planned analysis concerned the subgroup of children with chronic diseases. The primary research question concerned the range of indications for AM therapy as well as the outcome of disease symptoms. Further research questions concerned quality of life, use of adjunctive non-AM therapies, therapy satisfaction, and adverse reactions.

### Setting, participants, and therapy

All physicians certified by the Physicians' Association for Anthroposophical Medicine in Germany and working in an office-based practice or outpatient clinic were invited to participate in the AMOS study. Certification as an AM physician required a completed medical degree and a three-year structured postgraduate training. The participating physicians recruited consecutive patients starting AM therapy under routine clinical conditions. Patients enrolled in the period 1 January 1999 to 31 December 2005 were included in the present analysis if they fulfilled the eligibility criteria. Inclusion criteria were:

1. Outpatients aged 1–16 years.

2. AM-related consultation of at least 30 minutes followed by prescription of AM medication or referral to AM therapy (art, eurythmy or rhythmical massage) for any indication (main diagnosis).

3. Duration of main diagnosis of at least 30 days at study enrolment.

Patients were excluded if they had previously received the AM therapy in question (see inclusion criteria no. 2) for their main diagnosis. AM therapy was evaluated as a whole system [48] with subgroup analysis of evaluable therapy modality groups.

### Primary outcome

Primary outcome was disease severity at six-month follow-up. Disease severity was assessed on numerical rating scales [49] from 0 („not present“) to 10 („worst possible“): Disease Score (physicians' global assessment of severity of main diagnosis); Symptom Score (caregivers' assessment of severity of one to six most relevant symptoms present at baseline). Disease Score was documented after 0 and 6 months, Symptom Score and quality of life (see below) after 0, 3, 6, 12, 18, and 24 months.

### Secondary outcomes

In patients aged 8–16 years, quality of life was assessed with self-report, using the KINDL^® ^Questionnaire for Measuring Health-Related Quality of Life in Children and Adolescents, Total Quality of Life Score (0–100). For patients enrolled up till March 2001 the KINDL 40-item version [50] was used; for patients enrolled April 2001 and thereafter the KINDL 24-item version [51] (Kid-KINDL^® ^for age 8–12 years, Kiddo-KINDL^® ^for age 13–16 years) was used. The KINDL questionnaire addresses physical and emotional well-being, self-esteem, family, friends, and everyday functioning.

In patients aged < 8 years, quality of life was assessed by caregivers. For patients enrolled up till March 2001 the KITA Quality of Life Questionnaire [52] (age 1–7 years) was used. The KITA questionnaire comprises the subscales Psychosoma and Daily Life (0–100). For patients enrolled April 2001 and thereafter Kiddy-KINDL^®^, Total Quality of Life Score [51] (age 4–7 years) was used.

Therapy outcome rating (0–10), satisfaction with therapy (0–10), and therapy effectiveness rating ("very effective, "effective", "less effective", "ineffective" or "not evaluable") were documented by the caregivers (effectiveness rating also by the physicians) after 6 and 12 months. Adverse reactions to medications or therapies were documented by the caregivers after 6, 12, 18, and 24 months, and by the physicians after 6 months (for patients enrolled before 1 April 2001 also after 3, 9, and 12 months). The documentation included suspected cause, intensity (mild/moderate/severe = no/some/complete impairment of normal daily activities), and therapy withdrawal because of adverse reactions. Serious adverse events (death, life-threatening condition, acute in-patient hospitalization, new disease or accident causing permanent disability, congenital anomaly, new malignancy) were documented by the physicians throughout the study.

### Data collection

All data were documented with questionnaires returned in sealed envelopes to the study office. The physicians documented eligibility criteria; the therapists documented AM therapy administration; all other items were documented by the caregivers or patients unless otherwise stated. The patient responses were not made available to the physicians. The physicians were compensated 40 Euro (after March 2001: 60 Euro) per included and fully documented patient, while the patients and their caregivers received no compensation.

The data were entered twice by two different persons into Microsoft^® ^Access 97. The two datasets were compared and discrepancies resolved by checking with the original data.

### Quality assurance, adherence to regulations

The study was approved by the Ethics Committee of the Faculty of Medicine Charité, Humboldt University, Berlin, Germany, and was conducted according to the Declaration of Helsinki and largely following the ICH Guideline for Good Clinical Practice E6. Written informed consent was obtained from all patients before enrolment.

### Data analysis

The data analysis was performed on all patients fulfilling the eligibility criteria, using SPSS^® ^14.0.1 (SPSS Inc., Chicago, Ill, USA) and StatXact^® ^5.0.3 (Cytel Software Corporation, Cambridge, MA, USA). Diagnoses were coded according to the International Classification of Diseases, Tenth Revision (ICD-10).

For continuous data the two-tailed t-test was used. For binominal data the two-tailed McNemar test and Fisher's exact test were used. Significance criteria were p < 0.05 or 95% confidence interval (95%-CI) not including 0. Since this was a descriptive study, no adjustment for multiple comparisons was performed [53].

Pre-post effect sizes were calculated as Standardised Response Mean (= mean change score divided by the standard deviation of the change score) and classified as minimal (< 0.20), small (0.20–0.49), medium (0.50–0.79), and large (≥ 0.80) [54,55]. In the main analysis, clinical outcomes were analysed in patients with evaluable data for each follow-up, without replacement of missing values.

Three pre-planned sensitivity analyses (SA1–SA3) were performed to assess the influence of patient attrition, natural recovery, and adjunctive diagnosis-relevant non-AM therapies on the 0–6-month Symptom Score outcome. SA1 concerned attrition bias: Missing values after six months were replaced with the last value carried forward. SA2 concerned natural recovery, which was assumed to be unlikely in AMOS patients with disease duration of at least one year [56]: The sample was therefore restricted to patients with disease duration of at least 12 months prior to study enrolment. SA3 concerned the effects of relevant non-AM therapies, and was performed on diagnosis groups with at least 15 evaluable patients (mental, respiratory or musculoskeletal diseases, headache syndromes, urinary incontinence). In SA3 this sample was restricted to patients not using diagnosis-related non-AM therapies during the first six study months (listed in Table 1).

**Table 1:** Diagnosis-related non-anthroposophic therapies in months 0–6

**Main diagnosis (International Classification of Diseases, Tenth Edition)**	**Non-anthroposophic therapies**		**Patients with therapy**	
	**Drugs (Anatomical Therapeutic Chemical Classification Index)**	**Non-medication**	**N**	**%**

Mental disorders (F00–F99)	Antiepileptic, psycholeptic, analeptic, and anti-addiction drugs (N03A, N05–06, N07B)	Occupational therapy, play therapy	31/175	17.7%

Respiratory disorders (J00–J99)	Respiratory drugs (H02, J01–02, J04–05, J07A, L03, R01, R03, R06–07),	Relevant surgery	18/52	34.6%

Musculoskeletal diseases (M00–M99)	Immunosuppressive, musculoskeletal, analgesic, and antidepressant drugs (L04, M01–05, M09, N02A-B, N06A)	Physiotherapy, relevant surgery	3/16	18.8%

Headache disorders (G43–G44, R51)	Analgesics, antimigraine drugs, antidepressants (C04AX01, C07AA05, C07AB02, C08CA06, C08DA01, N02, N03AG01, N06A, N07CA03)		0/17	0.0%

Urinary incontinence (R32)	Vasopressin and analogues (H01BA)	Alarm therapy, occupational therapy, play therapy	1/36	2.8%

Total (evaluable patients)			53/296	17.9%

Stepwise multiple linear regression analysis was performed to identify predictors of Symptom Score change from baseline after 6 and 12 months. Criterion for inclusion of variables in the model was p < 0.05 and for exclusion p ≥ 0.10. The following independent variables were analysed:

• Socio-demographics: age, gender, household size, living with father, visiting Waldorf School, health insurance coverage, year of enrolment.

• Disease status at baseline: diagnosis (five categories), disease duration, disease severity, baseline Symptom Score, number of comorbid disorders, therapies in the preceding year (number of AM therapy sessions, number of patient-months of AM medication and non-AM medications, respectively, number of sessions with physiotherapy/occupational therapy/play therapy).

• Therapy factors: physician setting (primary care or other), physician qualification (general practitioner, paediatrician), number of years since the physician's medical qualification, previous treatment by the physician, number of patients enrolled by the physician, duration of consultation with the physician at study enrolment, main AM therapy modality (eurythmy, art, rhythmical massage, medical), number of years since the AM therapist's qualification, reimbursement of costs of AM therapies, therapies in months 0–6 (number of AM therapy sessions, number of patient-months of AM medication and non-AM medications, respectively, number of sessions with physiotherapy/occupational therapy/play therapy).

Missing values for independent variables were replaced by the respective mean values. Model assumptions for linear regression were checked and verified. A few outliers with studentised residuals ≥ 3 standard deviations were identified and excluded from the analyses (see Results for exceptions).

## Results

### Participating physicians and therapists

The patients were enrolled by 85 physicians with six different qualifications (57 general practitioners, 20 paediatricians, four internists, two otorhinolaryngologists, one gynaecologist, and one psychiatrist). Comparing these physicians to AM-certified physicians in Germany with the same six qualifications but without study patients (n = 295), no significant differences were found regarding gender (63.5% vs. 60.3% men), age (mean 46.6 ± 6.5 vs. 48.8 ± 8.3 years), number of years in practice (17.9 ± 7.1 vs. 19.8 ± 9.2 years) or the proportion of physicians working in primary care (88.2% vs. 83.7%).

The patients were treated by 131 different AM therapists (art, eurythmy, rhythmical massage). Comparing these therapists to certified AM therapists in Germany without study patients (n = 1,046), no significant differences were found regarding gender (78.6% vs. 81.1% women) or age (mean 49.4 ± 8.0 vs. 50.4 ± 9.6 years). The number of years since therapist qualification was 11.1 ± 6.4 and 13.4 ± 8.9 years in therapists with and without study patients, respectively (mean difference 2.3 years, 95%-CI 0.8–3.9 years, p = 0.004).

### Patient recruitment and follow-up

From 1 January 1999 to 31 December 2005, a total of 490 patients aged 1–16 years were assessed for eligibility. Of these patients, 435 fulfilled all eligibility criteria and were included in the study. 55 patients were not included for the following reasons: patients' baseline questionnaire missing (n = 15), physicians' baseline questionnaire missing (n = 8), patients' and physicians' baseline questionnaire dated > 30 days apart (n = 15), disease duration < 30 days (n = 13), previous or ongoing use of AM therapy in question (n = 4). Included and not included patients did not differ significantly regarding age, gender, disease duration, baseline Disease Score or baseline Symptom Score. A mental or behavioural disorder (ICD-10: F00–F99) was more frequent in included than in not included patients (46.2% vs. 25.5%, p = 0.004).

A total of 54.0% (235 of 435) of patients were enrolled by general practitioners, 41.4% by paediatricians, and 4.6% by other specialists. The physicians' settings were primary care practices (79.0% of evaluable patients, n = 323/409), referral practices (4.2%), and outpatient clinics (16.9%). Each physician enrolled 1–4 patients (71%, n = 60/85 physicians), 5–9 patients (20%) or ≥ 10 patients (13%), with a median of 2.0 patients enrolled per physician (range 1–62 patients, interquartile range [IQR] 1.0–5.5 patients).

The last patient follow-up ensued on 16 February 2008. A total of 97.5% (n = 424/435) of patients returned at least one follow-up questionnaire. The patients were administered a total of 2,175 follow-up questionnaires, out of which 1,816 (83.5%) were returned. Follow-up rates were 94.5% (n = 411/435), 88.3%, 83.7%, 77.2%, and 73.8% after 3, 6, 12, 18, and 24 months, respectively.

Respondents (n = 384) and non-respondents (n = 51) of the six-month patient-follow-up did not differ significantly regarding age, gender, diagnosis, disease duration, baseline Disease Score or baseline Symptom Score. Corresponding comparisons of respondents (n = 314) and non-respondents (n = 112) of the 24-month-follow-up also showed no significant differences, except for baseline Disease Score which was 6.4 ± 1.8 points in respondents and 6.9 ± 1.7 points in non-respondents (mean difference 0.6 points, 95%-CI 0.2–0.9 points, p = 0.005). The physicians' six-month follow-up documentation was available for 89.7% (n = 390/435) of patients.

### Baseline characteristics

The patients were recruited from 15 of 16 German federal states. Mean age was 8.2 ± 3.3 years (range 1.0–16.9 years). A total of 58.9% (n = 256/435) of the patients were boys. Mean household size (including the patient) was 4.2 ± 1.5 persons. A Waldorf school was attended by 50.4% (n = 139/276) of school pupils and by 32.0% of all patients.

The main diagnosis was a mental disorder (ICD-10: F00–F99) in 46% of patients (Table 2). Most common diagnosis subgroups were F90–F98 Behavioural and emotional disorders with onset usually occurring in childhood and adolescence (27.8%, n = 121/435 patients) and F80–F89 Disorders of psychological development (10.1%, n = 44). Most common three-digit level ICD-10 diagnoses were F90 Hyperkinetic disorders (16.1%, n = 70/435), R32 Unspecified urinary incontinence (8.7%, n = 38), J45 Asthma (8.0%, n = 35), and F98 Other behavioural and emotional disorders with onset usually occurring in childhood and adolescence (5.1%, n = 22). Median disease duration was 3.3 years (IQR 1.0–5.0 years, mean 3.4 ± 2.7 years).

**Table 2:** Baseline data of study population

**Item**	**N**	**%**
Age groups		

• 1–3 years	32/435	7.4%

• 4–7 years	183/435	42.1%

• 8–12 years	175/435	40.2%

• 13–16 years	45/435	10.3%

Living with		

• Mother	424/435	97.5%

• Father	339/435	77.9%

• Siblings	339/435	77.9%

• Other persons	50/435	11.5%

• More than one person	396/433	91.5%

School pupils		

• 1st to 3rd form	148/275	53.8%

• 4th to 6th form	86/275	31.3%

• 7th to 11th form	41/275	4.9%

Health insurance coverage		

• Statutory	389/434	89.6%

• Private	45/434	10.4%

Main diagnosis, International Classification of Diseases, Tenth Edition		

• F00–F99 Mental and behavioural disorders	201/435	46.2%

• R00–R99 Symptoms, signs and abnormal clinical and laboratory findings, not elsewhere classified	80/435	18.4%

• J00–J99 Diseases of the respiratory system	61/435	14.0%

• G00–G99 Diseases of the nervous system	25/435	5.7%

• M00–M99 Diseases of the musculoskeletal system and connective tissue	17/435	3.9%

• Other	51/435	11.7%

Disease duration		

• 1–2 months	15/435	3.4%

• 3–5 months	15/435	3.4%

• 6–11 months	40/435	9.2%

• 1–4 years	241/435	55.4%

• ≥ 5 years	124/435	28.5%

A current comorbid disease was present in 63.6% (n = 276/434) of evaluable patients, with a median of 1.0 (IQR 0.0–2.0) comorbid diseases. The most common comorbid diseases were R00–R99 Symptoms, signs and abnormal clinical and laboratory findings, not elsewhere classified (19.7%, n = 90/458 diagnoses), F00–F99 Mental and behavioural disorders (17.5%), J00–J99 Diseases of the respiratory system (13.8%), L00–L99 Diseases of the skin and subcutaneous tissue (9.6%), and D50–D89 Diseases of the blood and blood-forming organs and certain disorders involving the immune mechanism (6.1%). Further baseline data are presented in Table 2.

### Therapy

At study enrolment, the duration of the consultation with the AM physician was < 30 min in 53.1% (n = 231/435) of patients, 30–44 min in 21.1%, 45–59 min in 12.6%, and ≥ 60 min in 13.1% of patients. At enrolment, 16.3% (n = 71/435) of patients started AM therapy provided by the physician, while the remaining 83.7% (n = 364) were referred to AM eurythmy/art/massage therapy. Of these 364 patients, 86.8% (n = 316) had the planned AM therapy, 0.5% (n = 2) did not have AM therapy, and for 12.6% (n = 46) the AM therapy documentation is incomplete or inconclusive. AM therapies used were eurythmy therapy (n = 238 patients), rhythmical massage therapy (n = 29), and art therapy (n = 49) with the therapy modalities painting/drawing/clay (n = 17), speech exercises (n = 17), and music (n = 15). The AM eurythmy/art/massage therapy started median 13 (IQR 3–41) days after enrolment. Median therapy duration was 118 days (IQR 78–189 days), median number of therapy sessions was 12 (IQR 10–20). AM medications were used by 41.6% (n = 181/435) of patients in months 0–6 and by 69.2% (n = 301) in months 0–24.

Use of diagnosis-related non-AM therapies within the first six study months was analysed in patients with a main diagnosis of mental, respiratory or musculoskeletal diseases, headache syndromes (ICD-10 G43–G44, R51) or urinary incontinence, altogether n = 335 patients. Patients were classified as users if they had used at least one of the listed therapies (Table 1) for at least one day per month. Out of 296 evaluable patients, 82.1% (n = 243) had no diagnosis-related non-AM therapy.

### Primary outcome

**Figure 1:**
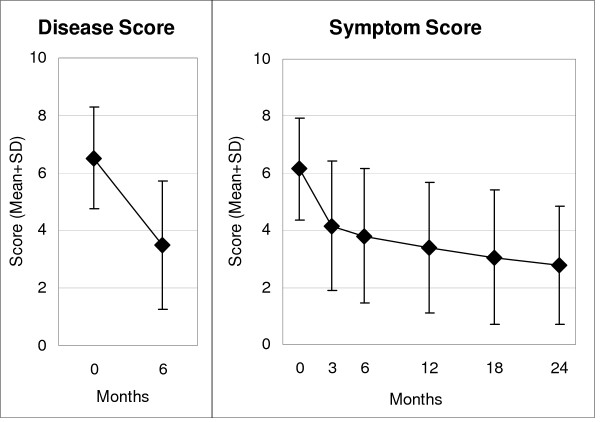
**Disease and Symptom Scores**. Range: 0 "not present", 10 "worst possible". Disease Score: physicians' assessment, n = 426. Symptom Score: caregivers' assessment, n = 433.

Disease and Symptom Scores (Figure 1) improved significantly and progressively between baseline and all subsequent follow-ups. After six months, an improvement of ≥ 50% of baseline scores was observed in 46.7% (n = 169/362) and 41.7% (n = 159/381) of evaluable patients for Disease and Symptom Scores, respectively. Standardised Response Mean effect sizes for the 0–6 month comparison were large for both scores (1.30 and 0.97, respectively). Symptom Score improved significantly in all four therapy modality groups (medical, eurythmy, art, rhythmical massage) (Table 3).

**Table 3:** Clinical outcomes 0–6 months

**Outcome (range)**	**Age years**	**N**	**0 months**	**6 months**	0–6 month difference*		**SRM**
			**Mean (SD)**	**Mean (SD)**	**Mean (95%-CI)**	**P-value**	

Disease Score (0–10)	1–16	362	6.49 (1.75)	3.49 (2.25)	3.00 (2.76–3.24)	< 0.001	1.30

Symptom Score (0–10)	1–16						

• All patients		381	6.21 (1.76)	3.80 (2.34)	2.41 (2.16–2.66)	< 0.001	0.97

• Medical treatment		60	5.72 (1.56)	2.86 (2.45)	2.86 (2.22–3.50)	< 0.001	1.16

• Eurythmy therapy		239	6.37 (1.84)	4.04 (2.33)	2.33 (2.02–2.65)	< 0.001	0.95

• Art therapy		51	6.35 (1.38)	3.86 (2.05)	2.49 (1.90–3.09)	< 0.001	1.18

• Rhythmical massage therapy		31	5.70 (1.84)	3.73 (2.32)	1.97 (0.82–3.11)	0.001	0.63

KINDL 40-item, Total** (0–100)	8–16	69	67.93 (11.18)	71.83 (11.18)	3.89 (1.43–6.36)	0.002	0.38

KINDL (Kid or Kiddo) Total*** (0–100)	8–16	104	68.48 (13.06)	72.19 (11.35)	3.71 (1.24–6.18)	0.004	0.29

KITA Psychosoma** (0–100)	1–7	80	67.92 (15.21)	75.73 (13.74)	7.81 (4.45–11.18)	< 0.001	0.52

KITA Daily Life** (0–100)	1–7	87	59.39 (19.48)	66.14 (19.03)	6.75 (3.00–10.50)	0.001	0.38

KINDL (Kiddy) Total*** (0–100)	4–7	104	67.82 (9.97)	71.78 (9.62)	3.97 (2.07–5.87)	< 0.001	0.41

*Positive differences indicate improvement. **Patients enrolled up till 31 March 2001. ***Patients enrolled 1 April 2001 and thereafter. SRM: Standardised Response Mean effect size (minimal: < 0.20, small: 0.20–0.49, medium: 0.50–0.79, large: ≥ 0.80).

We performed three sensitivity analyses of the 0–6 month outcome of Symptom Score (Table 4: SA1–SA3; see Methods for further description). SA1+2 had only small effects, reducing the average improvement by maximum 5% (2.41→2.28 points). SA3 increased the improvement by 9% (2.42→2.63 points). Combining SA1+2+3, Symptom Score improvement was increased by 5% (2.42→2.54 points).

**Table 4:** Symptom Score 0–6 months: sensitivity analyses (SA)

**Analysis**	**N**	**0 months**	**6 months**	**0–6 month difference**	
		**Mean (SD)**	**Mean (SD)**	**Mean (95%-CI)**	**P-value**

Main analysis: Patients with evaluable Symptom Score at 0 and 6 months	381	6.21 (1.76)	3.80 (2.34)	2.41 (2.16–2.66)	< 0.001

SA1: Last value carried forward	433	6.15 (1.80)	3.87 (2.35)	2.28 (2.05–2.52)	< 0.001

SA2: Patients with disease duration ≥ 12 months at study enrolment	320	6.29 (1.76)	3.91 (2.28)	2.39 (2.13–2.65)	< 0.001

SA1 + SA2	364	6.24 (1.78)	3.96 (2.30)	2.28 (2.03–2.52)	< 0.001

**Patients with main diagnosis of mental, musculoskeletal or respiratory diseases, headache disorders or urinary incontinence**					

Main analysis: Patients with evaluable Symptom Score at 0 and 6 months	297	6.26 (1.72)	3.84 (2.35)	2.42 (2.13–2.71)	< 0.001

SA3: Patients not using diagnosis-related non-anthroposophic therapies (see Table 1) in months 0–6	239	6.32 (1.71)	3.69 (2.29)	2.63 (2.32–2.95)	< 0.001

SA1 + SA2 + SA3	204	6.40 (1.71)	3.87 (2.21)	2.54 (2.22–2.86)	< 0.001

Predictors of Symptom Score improvement from baseline after 6 and 12 months were identified by stepwise multiple linear regression analysis (see Methods for details). The models for 0–6 month and 0–12 month improvement explained 23% and 26% of the variance, respectively (Table 5). Three variables were significant predictors in both models:

**Table 5:** Predictors of Symptom Score improvement: results of stepwise multiple linear regression analysis

**Variable**	**0–6 months (N = 380)**			**0–12 months (N = 360)**		
	**Adjusted R^2 ^change**	**B (95% confidence interval)**	**P-value**	**Adjusted R^2 ^change**	**B (95% confidence interval)**	**P-value**

Intercept		0.31 (-1.21 to 1.82)	0.690		0.67 (-0.57 to 1.90)	0.290

Symptom Score at enrolment (0–10)	0.17	0.67 (0.54 to 0.81)	< 0.001	0.20	0.71 (0.58 to 0.84)	< 0.001

Disease Score at enrolment (0–10)	0.03	-0.18 (-0.32 to -0.05)	0.007	0.02	-0.21 (-0.34 to -0.08)	0.002

Main AM therapy modality medical*	0.01	0.81 (0.20 to 1.42)	0.010	---	---	---

Previous treatment by physician*	0.01	0.60 (0.11 to 1.09)	0.016	---	---	---

Disease duration (years)	0.01	-0.10 (-0.19 to -0.02)	0.018	0.02	-0.14 (-0.23 to -0.05)	0.002

Physiotherapy, occupational therapy or play therapy in months 0–6 (sessions)	0.01	-0.03 (-0.05 to -0.003)	0.029	---	---	---

Main diagnosis F00–F99 Mental and behavioural disorder*	---	---	---	0.02	-0.65 (-1.08 to -0.22)	0.003

Enrolment by general practitioner*	---	---	---	0.01	0.58 (0.14 to 1.02)	0.010

	Total R^2 ^0.23			Total R^2 ^0.26		

**Analysis of variance for model**		**F-value**	**P-value**		**F-value**	**P-value**

		19,727	< 0.001		26,641	< 0.001

Adjusted R^2^: Adjusted coefficient of multiple determination. B: regression coefficient. *: 0 = no, 1 = yes. ---: Variable not included in the model.

• Symptom Score at baseline: For each 1.00 point increase in baseline Symptom Score (increase means worse symptoms), the Symptom Score improvement will increase by average 0.67 and 0.71 points after 6 and 12 months, respectively. Baseline Symptom Score was the strongest predictor, explaining 17% and 20% of the variance after 6 and 12 months, respectively.

• Disease Score at baseline: For each 1.00 point increase in baseline Disease Score (increase means more severe disease), the Symptom Score improvement will decrease by average 0.18 points and 0.21 points after 6 and 12 months, respectively.

• Disease duration: For each year of disease duration prior to study enrolment, the improvement will decrease by 0.10 and 0.14 points after 6 and 12 months, respectively.

In addition, the 0–6 month improvement was positively predicted by the variables 'main AM therapy modality medical' and 'previous treatment by physician' (these two predictions disappeared when one identified outlier was included in the analysis, see Methods for details) and negatively predicted by the number of sessions with physiotherapy, occupational therapy or play therapy in the previous year, while the 0–12 month improvement was positively predicted by enrolment by a general practitioner and negatively predicted by a main diagnosis of F00–F99 Mental and behavioural disorders.

### Secondary outcomes

The quality of life scores in all age groups improved significantly between baseline and nearly all subsequent follow-ups (23 significant and 2 non-significant improvements in 25 pre-post comparisons). Effect sizes for the 0–6 month comparison were medium for KITA Psychosoma (0.52) and small for the remaining four scores (range 0.29–0.41, Table 3).

At six-month follow-up, the caregivers' average therapy outcome rating (numeric scale from 0 "no help at all" to 10 "helped very well") was 7.12 ± 2.56 and the caregivers' satisfaction with therapy (from 0 "very dissatisfied" to 10 "very satisfied") was 7.87 ± 2.27.

The caregivers' effectiveness rating of eurythmy, art or rhythmical massage therapy was positive ("very effective" or "effective") in 78.8% (n = 242/307) of evaluable patients who had started therapy, and negative ("less effective", "ineffective" or "not evaluable") in 21.2%. The physicians' effectiveness rating was positive in 79.7% (n = 274/344) and negative in 20.3%. From 6- to 12-month follow-up, caregiver satisfaction with therapy decreased by average 0.38 points (95%-CI 0.13–0.63, p = 0.003) whereas caregivers' therapy outcome rating, and caregivers' as well as physicians' effectiveness rating did not change significantly.

Adverse reaction to AM therapies were reported in 1.3% (n = 4/316) of therapy users (eurythmy therapy: n = 2, rhythmical massage therapy: n = 2). The intensity of these reactions was moderate (n = 3) or not documented (n = 1); the AM therapy was stopped due to adverse reactions in two patients (eurythmy therapy: n = 1, rhythmical massage therapy: n = 1). The frequency of reported adverse drug reactions was 2.3% (n = 7/301 users) for AM medications and 8.3% (n = 22/264 users) for non-AM medications (p = 0.002).

Serious adverse events occurred in five patients. Four patients were acutely hospitalised and one patient had permanent disability from a whiplash injury. None of these events were causally related to any therapy or medication.

## Discussion

This two-year prospective cohort study is the first large study of AM therapy for children with chronic disease performed in office-based settings. We aimed to obtain information on AM therapy under routine conditions in Germany and studied the disease spectrum and clinical outcomes in children aged 1–16 years starting AM treatment for chronic diseases. Most frequent indications were mental/behavioural disorders (primarily hyperkinetic, emotional, and developmental disorders) and respiratory diseases. Following AM therapy, statistically significant improvements of disease symptoms and quality of life were observed. The symptom improvement was similar in patients not using diagnosis-relevant adjunctive non-AM therapies. A strong positive predictor of improvement after 6 and 12 months was higher baseline symptom intensity (caregivers' rating); weaker and negative predictors were higher disease severity (physicians' rating) and longer disease duration. Adverse reactions to AM therapies and medications were infrequent and not serious.

Strengths of this study include a detailed assessment of the therapy setting and therapy-related factors, a long follow-up period, and a high representativeness due to the participation of 14% of eligible AM physicians and AM therapists in Germany. The participating physicians and therapists resembled eligible but not participating AM physicians and AM therapists with respect to demographic characteristics, and the included patients resembled not included patients regarding baseline characteristics (except for a possible over-representation of mental disorders). These features suggest that the study to a high degree mirrors contemporary AM use in outpatient settings.

A limitation of the study is the absence of a comparison group receiving conventional treatment or no therapy. Accordingly, for the observed improvements one has to consider several other causes apart from the AM treatment. We therefore conducted a sensitivity analysis of Symptom Score, estimating the influence of attrition bias, adjunctive non-AM therapies, and natural recovery. These three factors together explained only 5% of the improvement. According to a previous analysis from this research program [56], regression to the mean due to symptom fluctuation with preferential self-selection to therapy and study inclusion at symptom peaks explained up till 0.43 points (14%) of the improvement of Disease Score, which would correspond to approximately 18% of the Symptom Score improvement in the present analysis. Other possible confounders are psychological factors and non-specific effects. However, since AM therapy was evaluated as a whole system [48], the question of specific therapy effects vs. non-specific effects (placebo effects, context effects, physician-patient interactions, patient expectations etc.) was not an issue of the present analysis.

Since patients were treated by AM physicians who could possibly have an interest in AM therapy having favourable outcomes, study data were largely collected by the patients and not the physicians. Any bias affecting the physicians' documentation would not affect Symptom Score or quality of life, since these clinical outcomes were documented by the patients or caregivers.

This analysis assessed AM as a whole system [48], with subgroup analyses of major therapy modality groups. In the bivariate analyses, symptom improvement was more outspoken among patients receiving AM medical therapy and less outspoken in the rhythmical massage therapy group (Table 3), but this was not reliably confirmed in the multivariate predictor analyses. Other measures of variability of AM treatment, such as the duration of the initial consultation or the number of AM therapy sessions, did not predict symptom improvement.

The strongest predictor of symptom improvement was baseline symptom severity as rated by caregivers. This finding can have several causes, such as more room for improvement and more regression to the mean with higher score values, the hello-goodbye effect, and a higher patient motivation with therapies working better at higher symptom levels [55,56]. Baseline disease severity, rated by the physicians, predicted future improvement in the opposite direction than baseline symptoms (i. e. lower disease severity predicted more improvement while lower symptom intensity predicted less improvement). A possible explanation for this seeming paradox is that physicians incorporate medical knowledge about the patients' prognosis into their severity rating, while caregivers focus more strongly on the symptoms. – A limitation of the predictor analysis is that parental education and occupational levels as well as household income could not be assessed, since these items were not documented for children. In a corresponding predictor analysis in adult patients from the AMOS project, these factors were not associated with symptom improvement (Hamre et al, submitted for publication).

Previous studies have evaluated AM therapy for children with chronic disorders including anorexia nervosa [36,38], atopic diseases [37,43], hyperkinetic disorders [24,45], epilepsy [44], hepatitis B [39], and immune suppression with recurrent respiratory infections following radioactive exposure after the Chernobyl nuclear accident [40-42]. All these studies had some favourable outcomes; the three largest studies (range 79–125 AM patients) found high anorexia cure rates [36], reduced infection rates and normalisation of immune parameters in Chernobyl children [42], and successful epilepsy therapy without conventional anticonvulsive drugs [44]. Except for a pilot study with five patients [24], these studies were performed in inpatient hospitals [36-39] or outpatient clinics [40-45]. In accordance with these studies from secondary care, our study from a predominantly primary care setting showed significant improvements of mental/behavioural, respiratory, and other chronic diseases in children following AM treatment. The largest improvements (large effect sizes, half of patients improved by at least 50% of their baseline scores) were observed for the items which directly measure the conditions treated with AM, i. e. Disease and Symptom Scores.

This study also underlines the role of naturalistic outcome studies in the evaluation of complementary therapies and other complex therapy systems [57,58]. For such outcome studies, several features are called for [48,57-60], which are found in the present study: primary assessment of the whole therapy complex and secondary assessment of therapy components; recruitment of patients in the setting where they are usually treated (here: predominantly primary care); documentation of routine therapy practice while minimising distortion from experimental study conditions; widely used outcomes (here: numerical rating scales); long-term follow-up; and, especially in the case of single-arm studies, a systematic approach to assess and minimise bias.

## Conclusion

In this study, children under AM treatment for mental/behavioural, respiratory, and other chronic diseases had long-term reduction of disease severity and improvement of quality of life. Improvements were similar in patients not using adjunctive non-AM therapies. Although the pre-post design of the present study does not allow for conclusions about comparative effectiveness, study findings suggest that AM therapies may play a beneficial role in the long-term care of children with chronic illness.

## List of abbreviations

±: standard deviation; 95%-CI: 95% confidence interval; AM: Anthroposophic Medicine; AMOS: Anthroposophic Medicine Outcomes Study; ICD-10: International Classification of Diseases, Tenth Edition; IQR: interquartile range; KINDL: KINDL^® ^Questionnaire for Measuring Health-Related Quality of Life in Children and Adolescents; SA: Sensitivity analysis.

## Competing interests

Within the last five years HJH and GSK have received restricted research grants and CM has received lecture fees from the pharmaceutical companies Weleda and Wala, who produce AM medications. Otherwise all authors declare that they have no competing interests.

## Authors' contributions

HJH, CMW, GSK, SNW, and HK contributed to study design. HJH, AG, CM, and HK contributed to data collection. HJH and HK wrote the analysis plan, HJH and AG analysed data. HJH was principal author of the paper, had full access to all data, and is guarantor. All authors contributed to manuscript drafting and revision and approved the final manuscript.

## Pre-publication history

The pre-publication history for this paper can be accessed here:


